# Isotope-Filtered 4D NMR Spectroscopy for Structure Determination of Humic Substances**

**DOI:** 10.1002/anie.201503321

**Published:** 2015-06-02

**Authors:** Nicholle G A Bell, Adam A L Michalchuk, John W T Blackburn, Margaret C Graham, Dušan Uhrín

**Affiliations:** EastChem School of Chemistry, University of Edinburgh, Joseph Black BuildingDavid Brewster Rd, Edinburgh EH9 3FJ (UK); School of Geosciences, University of Edinburgh, Grant InstituteJames Hutton Road, Edinburgh EH9 3FE (UK)

**Keywords:** complex mixtures, humic substances, isotopic labeling, NMR spectroscopy

## Abstract

Humic substances, the main component of soil organic matter, could form an integral part of green and sustainable solutions to the soil fertility problem. However, their global-scale application is hindered from both scientific and regulatory perspectives by the lack of understanding of the molecular make-up of these chromatographically inseparable mixtures containing thousands of molecules. Here we show how multidimensional NMR spectroscopy of isotopically tagged molecules enables structure characterization of humic compounds. We illustrate this approach by identifying major substitution patterns of phenolic aromatic moieties of a peat soil fulvic acid, an operational fraction of humic substances. Our methodology represents a paradigm shift in the use of NMR active tags in structure determination of small molecules in complex mixtures. Unlike previous tagging methodologies that focused on the signals of the tags, we utilize tags to directly probe the identity of the molecules they are attached to.

The world’s population is estimated to rise to 11 billion by 2100,[1] putting unparalleled pressure on agricultural food production.[2] New sustainable means of increasing land fertility whilst avoiding the current overuse of NPK fertilizers are needed.[2c, 3] Humic substances, which exist in soil as complex mixtures of thousands of organic compounds derived from animal and plant remains, could form an integral part of green and sustainable solutions to the soil fertility problem. Humic substances play a vital role in physical and biochemical soil functions,[4] stimulate plant growth,[5] and ameliorate the effect of contaminant metals and chemical residues, which may build up over time and hinder plant growth.[6] It is unclear, however, if all fractions of humic substances are beneficial to plant growth,[5c] and if certain humic molecules may indeed be harmful to humans.[7] Thus, to address the potential role of humic substances in restoring the fertility of intensively farmed agricultural soils, it is essential to determine their molecular composition. Such an achievement will allow development of a molecular rather than a phenomenological description of soil—a fundamental step toward defining the structure–function relationships of humic substances. Nevertheless, the two high-resolution analytical techniques, Fourier transform ion cyclotron resonance mass spectrometry (FT ICR MS)[8] and NMR spectroscopy,[6b, 9] which are essential in this endeavor, have serious shortcomings: MS only provides molecular formulae, and standard 2D or 3D NMR experiments cannot deliver the structures of individual compounds contained in complex mixtures.

The development of more powerful multidimensional (*n*D) NMR experiments is essential in overcoming these limitations.[10] However, *n*D NMR alone cannot address the inherent complexity of chromatographically inseparable mixtures,[10a] and some form of “spectroscopic separation” is still necessary. To achieve this, we have recently developed an isotope-filtered *n*D NMR methodology[11]—a combination of isotopic tagging and *n*D NMR. Unlike previous tagging approaches that focused on the signals of the tags,[6b, 12] we utilize them to probe directly the identity of the tagged molecules. The power of this approach is in the ability to provide multiple correlated chemical shifts of individual molecules.

Here we report how isotope-filtered *n*D NMR enables characterization of phenolic moieties of humic molecules and illustrate its principles using a 4D ^13^CH_3_O-filtered NMR experiment, 4D HCCH_3_, which correlates chemical shifts of four nuclei—the aromatic CH atoms *ortho* to methoxy groups and those of ^13^CH_3_O atoms. The polarization transfer pathway of this experiment starts on aromatic protons and finishes on methyl protons utilizing ^1^*J*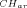, ^3^*J*_CC_, and ^1^*J*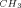 couplings (Figure 1 a). The pulse sequence of 4D HCCH_3_ is shown in Figure S1 in the Supporting Information.

**Figure 1:**
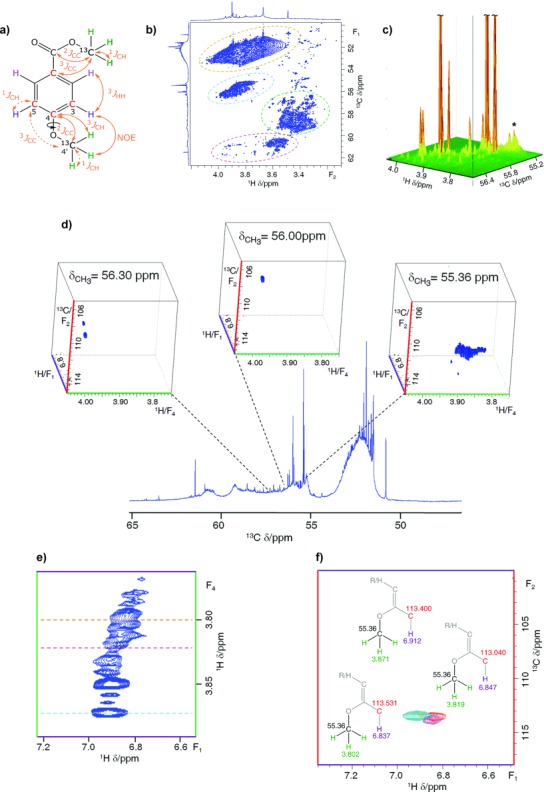
a) An exemplar aromatic compound highlighting nuclei accessible by ^13^CH_3_O-filtered *n*D NMR experiments. The interactions mediating the polarization transfers in the 4D HCCH_3_ are shown as dashed double-headed arrows. The color coding of nuclei is the same as used for the chemical shift axes of *n*D NMR spectra; b) the methoxy region of the 800 MHz 2D ^1^H,^13^C HSQC spectrum of ^13^CH_3_O-methylated FA. Circled areas identify subregions as ester (orange), phenolic (cyan), carbohydrate (green), and aromatic/aliphatic sterically hindered methoxy groups (magenta); c) A stack plot of the phenolic methoxy resonances corresponding to the cyan-circled cross peaks in (b). The region of the spectrum used to illustrate the 4D HCCH_3_ experiment below is labeled with an asterisk. d) Exemplar 3D cuboids extracted from a 800 MHz 4D HCCH_3_ spectrum of ^13^C-methylated FA at ^13^C(H_3_) chemical shifts indicated by the dashed lines. e) An F_1_F_4_ (H_ar_(C)H_3_) projection of a cuboid extracted at 55.36 ppm; f) An overlay of three 2D F_1_F_2_ (H_ar_C_ar_) planes extracted from this cuboid at proton methoxy chemical shifts indicated by the colored dashed lines in (e). Insets show the identified structural fragments, which belong to compounds 23, 32, and 33 (Figure S2).

The workings of this experiment are illustrated using a peat soil fulvic acid (FA) sample that was methylated using ^13^CH_3_I.[11] The sample was first characterized by acquiring a 2D ^1^H,^13^C HSQC spectrum (Figure 1 b). Its methoxy region shows a spread of ^13^CH_3_ cross peaks reflecting different chemical environments of the methoxy groups. As the 4D HCCH_3_ experiment was designed to investigate phenolic compounds, an expansion of the phenolic methoxy region of the 2D ^1^H,^13^C HSQC spectrum is shown as a stacked plot in Figure 1 c. This presentation reveals the presence of several major and numerous minor phenolic compounds in this FA sample.

A partial 4D HCCH_3_ spectrum of ^13^C-methylated FA shown in Figure 1 d illustrates how isotope filtration combined with the dispersion of signals in a 4D space leads to comprehensive structural information. A cuboid extracted at 55.36 ppm is inspected. This region of the spectrum contains severely overlapping medium intensity signals labeled with an asterisk on Figure 1 c. An F_1_F_4_ (or H_ar_(C_ar_COC)H_3_) projection of the 55.36 ppm cuboid shows an adequate spread of signals in the directly detected F_4_ dimension (Figure 1 e). An overlay of three 2D F_1_F_2_ (or H_ar_C_ar_) planes taken from this cuboid at methyl proton chemical shifts indicated by the dashed lines in Figure 1 e reveals similar, but non-identical, H_ar_/C_ar_ chemical shifts (Figure 1 f). The inspected region of the 4D HCCH_3_ spectrum thus provides unprecedented chemical shift separation of aromatic compounds which have identical aromatic ring substitution patterns, whereas their substituents show structural variations further away from the aromatic ring.

Additional 3D ^13^CH_3_O-filtered experiments were designed to complement the 4D experiment. 3D variants of the 4D HCCH_3_, 3D H(C)CH_3_, and 3D (H)CCH_3_, do not label aromatic carbon or proton resonances, respectively, and hence provide similar, but more ambiguous information than the 4D spectra albeit with higher digital resolution. Chemical shifts of nuclei other than those *ortho* to the ^13^CH_3_O groups (Table S1), were obtained by 3D INEPT-INADEQUATE-HSQC[11]/3D C_q_CH_3_ (quaternary aromatic carbons) and 3D CH_3_-NOESY/3D CH_3_-NOESY-TOCSY (*meta* and *para* protons relative to ^13^CH_3_O groups). These NMR experiments utilize polarization transfer through various couplings shown in Figure 1 a. The NOESY- and TOCSY- based experiments also show the splitting of aromatic proton resonances due to ^3^*J*_HH_ or ^4^*J*_HH_ couplings, hence providing additional valuable structural information.

In summary, multiple chemical shifts and coupling constants were obtained for individual molecules by analyzing the 3D/4D ^13^CH_3_O-filtered spectra. When cross-referenced against database information,[13] this lead to the identification of nine major phenolic compounds of this FA sample (Figure 2). These comprise 1,3,4-, 1,3,5-trisubstituted as well as 1,4-disubstituted hydroxybenzenes that differ by the nature of the *para* substituent relative to the ^13^CH_3_O group.

**Figure 2:**
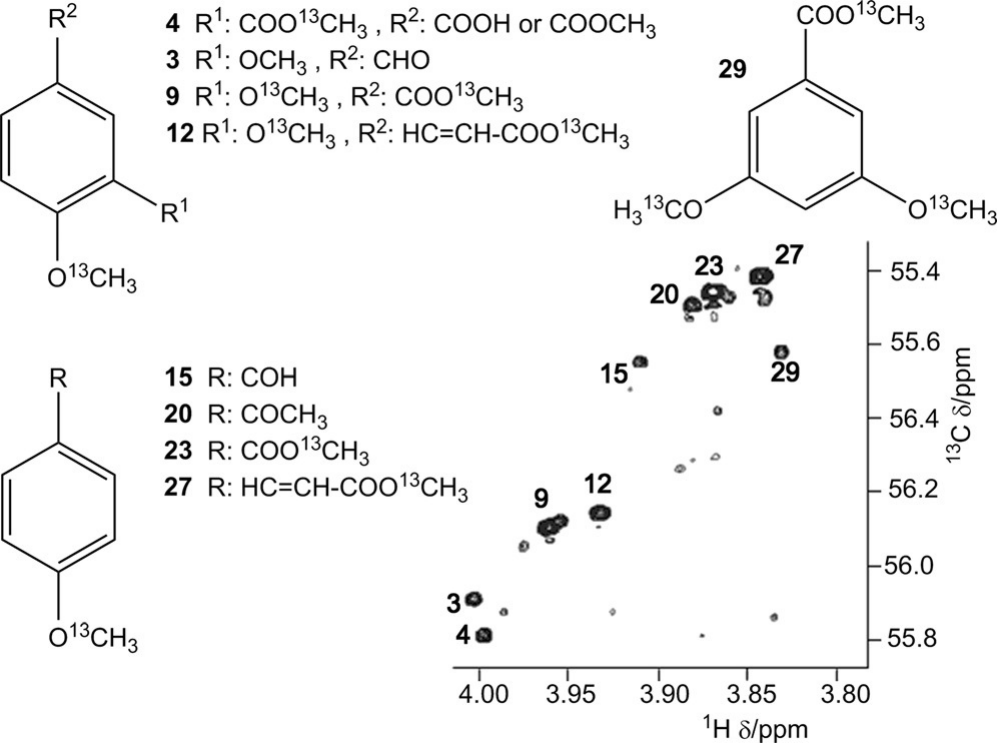
Major phenolic compounds identified in the methylated FA sample; their methoxy cross peaks are labeled on the 2D ^1^H,^13^C HSQC spectrum.

Twenty-one additional structures/structural motifs were also identified (Figure S2). Some of these contained highly substituted aromatic rings with four or five substituents. These molecules mainly relate to plant-derived lignin precursors such as vanillin, coumaryl, syringyl, coniferyl monolignols, but also flavonoids. The presence of several of these molecules in humic substances has been postulated based on the results of tetramethyl ammonium hydroxide (TMAH)-assisted pyrolysis GC-MS analysis.[14] Aromatic ring substitution patterns identified in our work were previously suggested based on the bulk matching of experimental and theoretical 2D ^1^H,^13^C HSQC spectra; the latter were calculated by considering the effects of -OR and -COOR groups on the ^1^H and ^13^C chemical shift of CH atoms.[15] These approaches relied on matching a limited number of descriptors to numerous possibilities. In contrast, our methodology provides a multitude of correlated chemical shifts, which allows convergence to a single structure/structural motif, thus yielding unprecedented structural details for phenolic compounds in samples of humic substances.

Further advances of this methodology are not limited to methylation. In addition, other tags containing NMR-active nuclei such as ^15^N, ^19^F, and ^31^P, represent promising candidates for tagging various functional groups. Once fully developed, this methodology will lay ground for the structure–function investigations of humic compounds thus enabling exploration of their roles in improving the soil fertility and sustainable food production. Isotope-filtered *n*D NMR spectroscopy is also applicable to the investigation of natural organic matter in general, but also complex mixtures other than those found in the environment, for example, small-molecular metabolites, plant extracts, oil, food, and beverages.

## Experimental Section

Peaty soil was collected from the Red Moss, an ombrotrophic peat bog near Balerno, central Scotland. The FA sample (60 mg) was extracted using the International Humic Substance Society (IHSS) protocols (http://www.humicsubstances.org/isolation.html) and methylated as described previously[11] yielding ^13^C-methylated FA (10 mg). The sample was dissolved in 550 μL of CDCl_3_ for NMR analysis at 15 °C. The pulse sequence of the 4D HCCH_3_ is shown in Figure S1. The parameters of NMR experiments are detailed in the Supporting Information.

## Supporting Information

As a service to our authors and readers, this journal provides supporting information supplied by the authors. Such materials are peer reviewed and may be re-organized for online delivery, but are not copy-edited or typeset. Technical support issues arising from supporting information (other than missing files) should be addressed to the authors.

miscellaneous_information
