# Supplementary material for: Isotope-Filtered 4D NMR Spectroscopy for Structure Determination of Humic Substances
Source: Angew Chem Int Ed Engl. 2015 Jun 2;54(29):8382–5. doi: 10.1002/anie.201503321 (PMC4531828; doi:10.1002/anie.201503321)
Supplement: Supplementary file 1 — miscellaneous_information [file anie0054-8382-sd1.pdf]

Angewandte Chemie
Eine Zeitschrift der Gesellschaft Deutscher Chemiker

Supporting Information

Isotope-Filtered 4D NMR Spectroscopy for Structure Determination of Humic Substances**

*Nicholle G. A. Bell, Adam A. L. Michalchuk, John W. T. Blackburn, Margaret C. Graham, and Dušan Uhrín**

anie_201503321_sm_miscellaneous_information.pdf

Supporting Information

Content:

Table 1
Figure S1
NMR parameters
Figure S2

Table 1. $^{13}CH_3O$**-filtered nD NMR experiments and the chemical shifts they provide**[a]**.**

Experiment/Dimension	F_1	F_2	F_3	F_4
4D $HCCH_3$	H_{Ar}	C_{Ar}	$C(H_3)$	$(C)H_3$
3D $H(C)CH_3$	H_{Ar}	$C(H_3)$	$(C)H_3$	-
3D $(H)CCH_3$	C_{Ar}	$C(H_3)$	$(C)H_3$	-
3D INEPT-INADEQUATE-HSQC	$[C_{Ar}C(H_3)]_{DQ}$	$C(H_3)/C_{Ar}$	$(C)H_3/H_{Ar}$	-
3D C_qCH_3	$C(H_3)$	C_{Ar}	$(C)H_3$	-
3D CH_3-NOESY	$(C)H_3$	$C(H_3)$	$H_{Ar\text{-}ortho}$	-
3D CH_3-NOESY-TOCSY	$(C)H_3$	$C(H_3)$	$H_{Ar\text{-}meta}$	-

[a] The chemical shifts of nuclei enclosed in parentheses are not labelled.

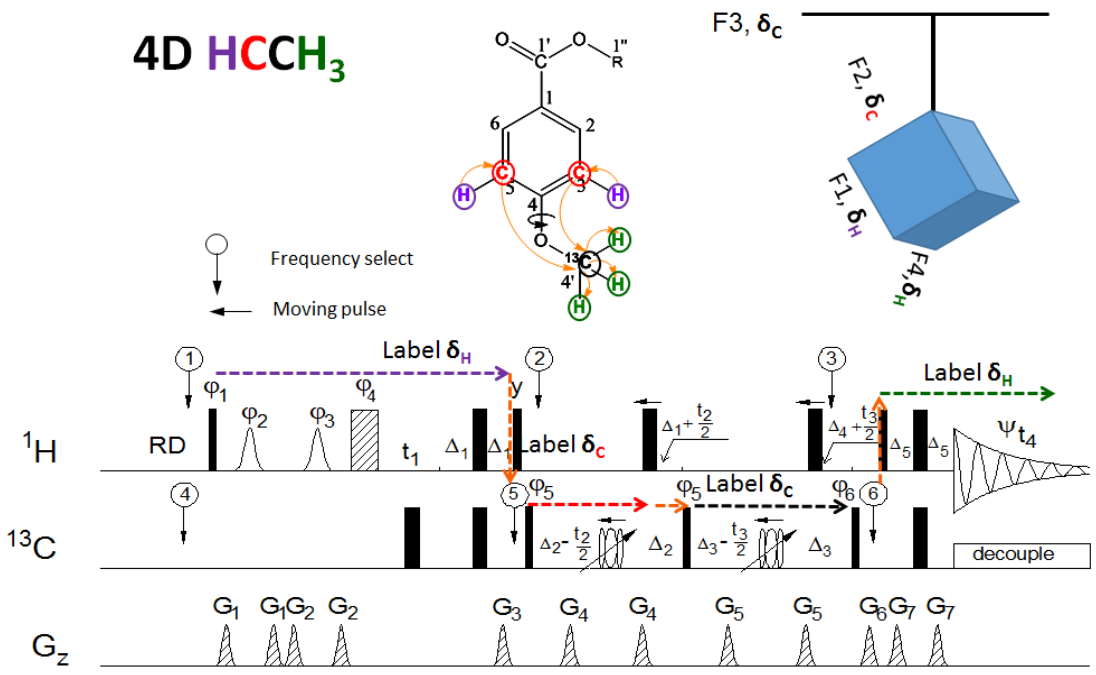

Fig S1. Pulse sequence of the 4D $HCCH_3$ experiment. The polarization transfers pathway is labelled by colour coded dashed lines corresponding to the labelling of nuclei on the structure shown in the inset. This colour coding is adhered also in the Fig. 1 and 2 of the main text, as well as Fig. S2. The inset also shows the way the four dimensions were ordered when inspecting the spectra. The thin and thick bar represent 90° and 180° pulses, respectively, if not stated otherwise applied from the x axis. The shaped ^{1}H pulses were 1 ms, rSNOB pulses (E. Kupce, J. Boyd, I.D. Campbell, *J. Magn. Reson. B* **106**, 300-303 (1995)) and the dashed ^{1}H pulse was a 1 ms rectangular trim pulse applied at full power. Two 2 ms composite ^{13}C CHIRP pulses were used in the middle of $2\Delta_2$ and $2\Delta_3$ intervals. To achieve pure phase of aromatic proton resonance in F_1, the pulse sequence starts with a DPFGSE (T.L. Hwank and A.J. Shaka *J. Magn. Reson. A* **112**, 275-279 (1995)) rather than a 90° selective excitation pulse. The DPFGSE also provides much better initial suppression of resonances outside of the inverted region. The following phase cycling was used: φ_1 = x; φ_2 = 4x, 4y; φ_3 = 2x, 2y; φ_4 = y; φ_5 = x, -x; φ_6 = x; ψ=x,-x. The delays were set as $\Delta_1 = 1/4^1J_{CHar}$ (155 Hz), $\Delta_2 = 1/4^nJ_{CC}$ (6 Hz), $\Delta_3 = 1/4^nJ_{CC}$ (8 Hz), $\Delta_4 = 0.915/^1J_{CH3}$ (145 Hz), $\Delta_5 = 1/4^1J_{CH3}$ (145 Hz). The 1 ms PFGs were applied at G_1= 33%, G_2 = 93%, G_3= 23%, G_4= 8%, G_5= 17%, G_6= 33%, G_7= 10% of the maximum strength. The frequency of the ^{1}H and ^{13}C channels were changed at indicated time points as follows: 1: 7.5 ppm, 2: 5.52 ppm, 3: 3.8 ppm, 4: 110 ppm, 5: 96 ppm and 6: 56 ppm. The φ_1, φ_4, and φ_5 and φ_6 phases were incremented according to the States-TPPI protocol. In addition, when t_1 delay was incremented, the φ_1, φ_2 and φ_4 were incremented by -20°. When t_2 delay was incremented, φ_5 was incremented by +135°. This resulted in spectral width dependent displacement of resonances within the set spectral widths. Together with folding of resonances this allowed the minimization of spectral widths and maximized the digital resolution.

NMR parameters

1D ^{13}C NMR spectrum (Figure 1d) was obtained using acquisition and relaxation times of 0.63 and 2s; 128 scans were accumulated. The ^{1}H, ^{13}C 2D HSQC spectrum (Figure 1b,c) was recorded using Bruker *hsqctgp* pulse sequence. The delays were optimised for a $^1J_{CH}$ coupling of 145 Hz. 6144 x 1024 TD points were acquired during t_2 and t_1 acquisition times of 320 and 127 ms, respectively, covering 12 and 20 ppm. The relaxation delay was 2 s and two scans were acquired per each t_1 increment. The 4D $HCCH_3$ spectrum was recorded with 576 x 24 x 56 x 16 data points sampling spectral widths of 1.5, 4, 20, 1.2 ppm and yielding t_4, t_3, t_2, and t_1 acquisition times of 240, 15, 7 and 8 ms, respectively. Eight scans were accumulated into each increment. The relaxation delay was 0.9 s resulting in the overall acquisition time of 64 hours.

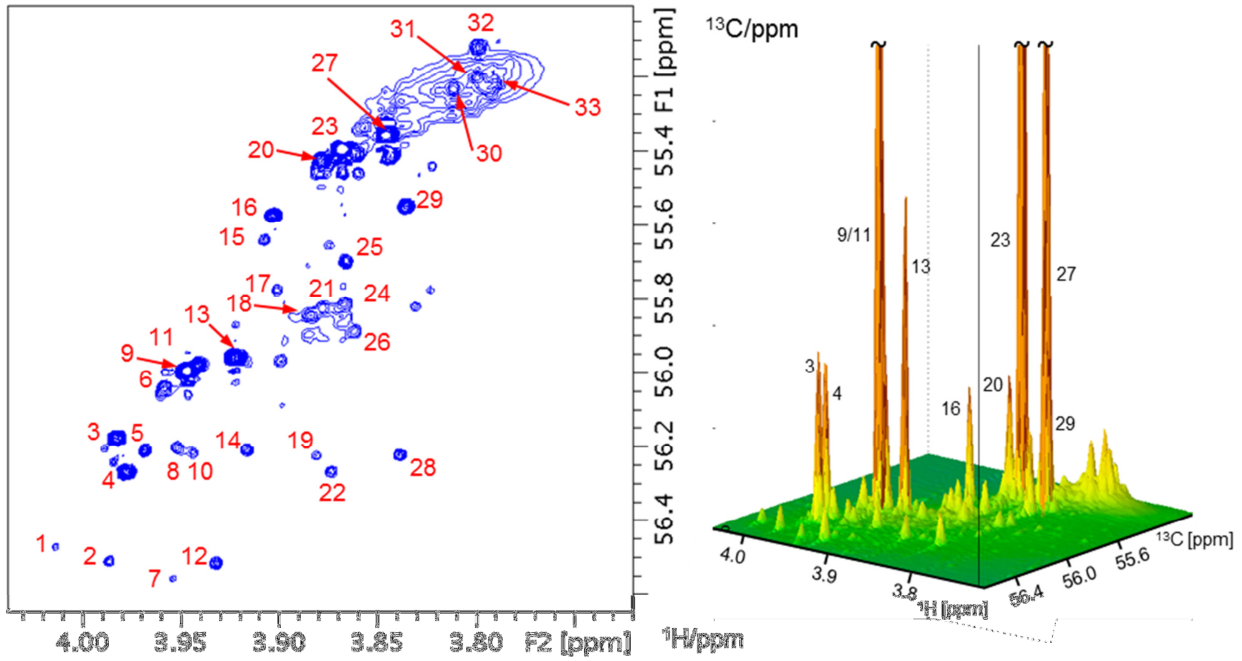

Fig. S2. The methoxy region of the 2D ^{1}H , ^{13}C HSQC spectrum of methylated FA shown as a contour (left) and a stacked (right) plot. The structures corresponding to the labelled cross peaks (1-33) are shown below. The chemical shifts (ppm) obtained from the analysis of ^{13}CH$_3$O- filtered nD NMR experiments are given in black; the database chemical shifts are shown in grey. The ^{13}CH$_3$ cross peak reference numbers are indicated inside of the aromatic rings. The colour coding of nuclei indicates different chemical shift axes of nD NMR spectra.

R_1, R_2: C(not O)
R_3: COCH=CHR

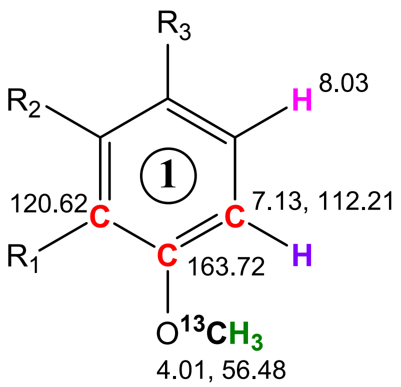

R_1, R_2, R_4: C (not O)
R_3: COCH=CHR

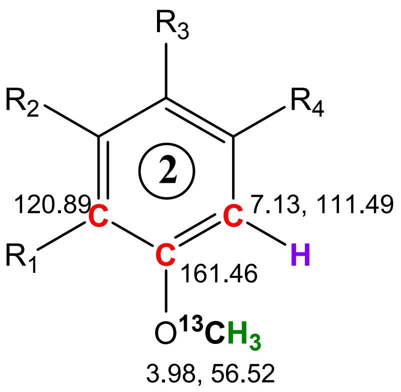

R_1, R_2, R_4: C (not O)
R_3: COCH=CHR

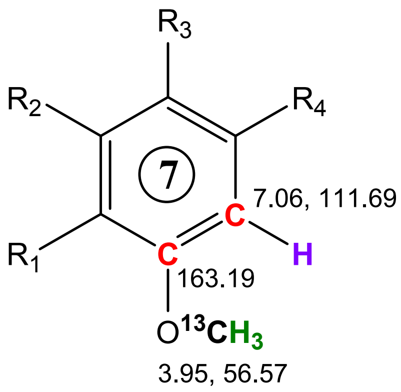

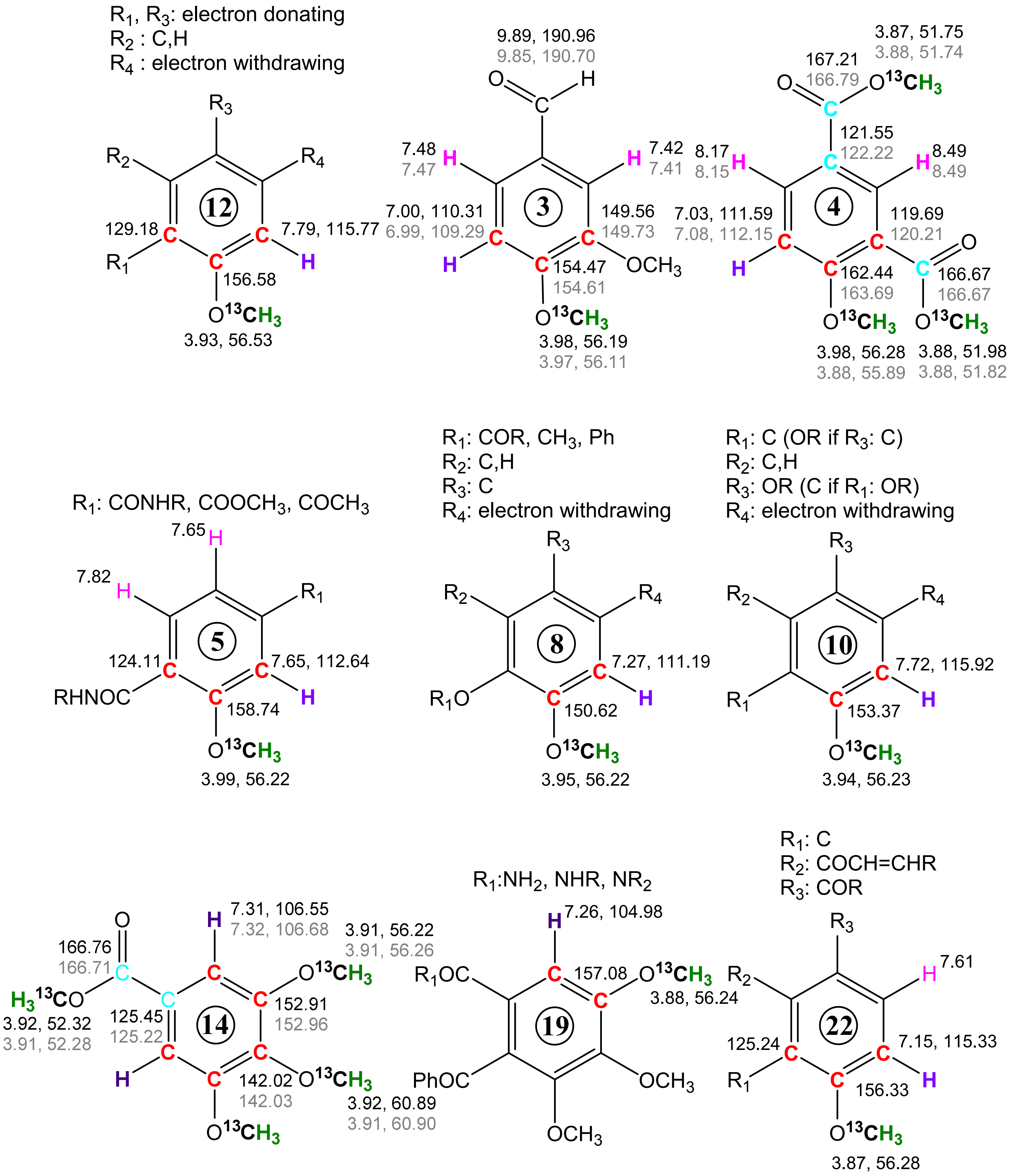

R1, R3: electron donating
R2 : C,H
R4 : electron withdrawing
12
129.18
7.79, 115.77
156.58
3.93, 56.53
3
9.89, 190.96
9.85, 190.70
7.48
7.47
7.42
7.41
7.00, 110.31
6.99, 109.29
149.56
149.73
154.47
154.61
OCH3
3.98, 56.19
3.97, 56.11
4
3.87, 51.75
3.88, 51.74
167.21
166.79
121.55
122.22
8.17
8.15
8.49
8.49
7.03, 111.59
7.08, 112.15
119.69
120.21
162.44
163.69
166.67
166.67
3.98, 56.28
3.88, 55.89
3.88, 51.98
3.88, 51.82
R1: CONHR, COOCH3, COCH3
5
7.65
7.82
124.11
7.65, 112.64
RHNOC
158.74
3.99, 56.22
R1: COR, CH3, Ph
R2: C,H
R3: C
R4: electron withdrawing
8
R1O
7.27, 111.19
150.62
3.95, 56.22
R1: C (OR if R3: C)
R2: C,H
R3: OR (C if R1: OR)
R4: electron withdrawing
10
7.72, 115.92
153.37
3.94, 56.23
14
166.76
166.71
H3 13CO
3.92, 52.32
3.91, 52.28
125.45
125.22
7.31, 106.55
7.32, 106.68
3.91, 56.22
3.91, 56.26
152.91
152.96
142.02
142.03
3.92, 60.89
3.91, 60.90
R1:NH2, NHR, NR2
19
7.26, 104.98
R1OC
157.08
3.88, 56.24
PhOC
OCH3
OCH3
R1: C
R2: COCH=CHR
R3: COR
22
7.61
125.24
7.15, 115.33
156.33
3.87, 56.28

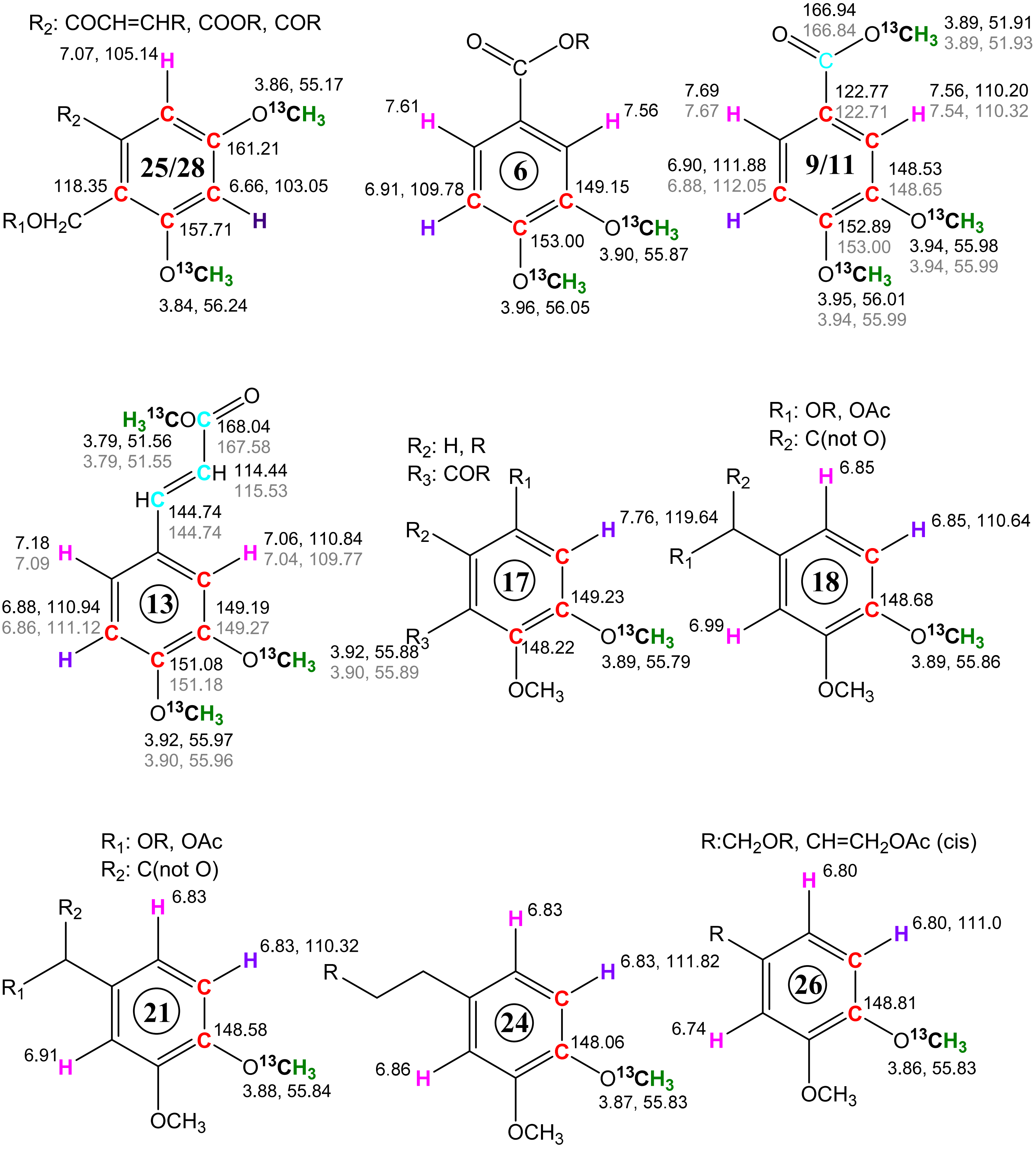

R2: COCH=CHR, COOR, COR
7.07, 105.14
3.86, 55.17
161.21
25/28
118.35
6.66, 103.05
R1OH2C
157.71
3.84, 56.24
OR
7.61
7.56
6
6.91, 109.78
149.15
153.00
3.90, 55.87
3.96, 56.05
166.94
166.84
3.89, 51.91
3.89, 51.93
7.69
7.67
122.77
122.71
7.56, 110.20
7.54, 110.32
6.90, 111.88
6.88, 112.05
9/11
148.53
148.65
152.89
153.00
3.94, 55.98
3.94, 55.99
3.95, 56.01
3.94, 55.99
168.04
167.58
3.79, 51.56
3.79, 51.55
114.44
115.53
144.74
144.74
7.18
7.09
7.06, 110.84
7.04, 109.77
6.88, 110.94
6.86, 111.12
13
149.19
149.27
151.08
151.18
3.92, 55.97
3.90, 55.96
R2: H, R
R3: COR
7.76, 119.64
17
149.23
148.22
3.92, 55.88
3.90, 55.89
3.89, 55.79
R1: OR, OAc
R2: C(not O)
6.85
6.85, 110.64
18
148.68
6.99
3.89, 55.86
R1: OR, OAc
R2: C(not O)
6.83
6.83, 110.32
21
148.58
6.91
3.88, 55.84
6.83
6.83, 111.82
24
148.06
6.86
3.87, 55.83
R:CH2OR, CH=CH2OAc (cis)
6.80
6.80, 111.0
26
148.81
6.74
3.86, 55.83

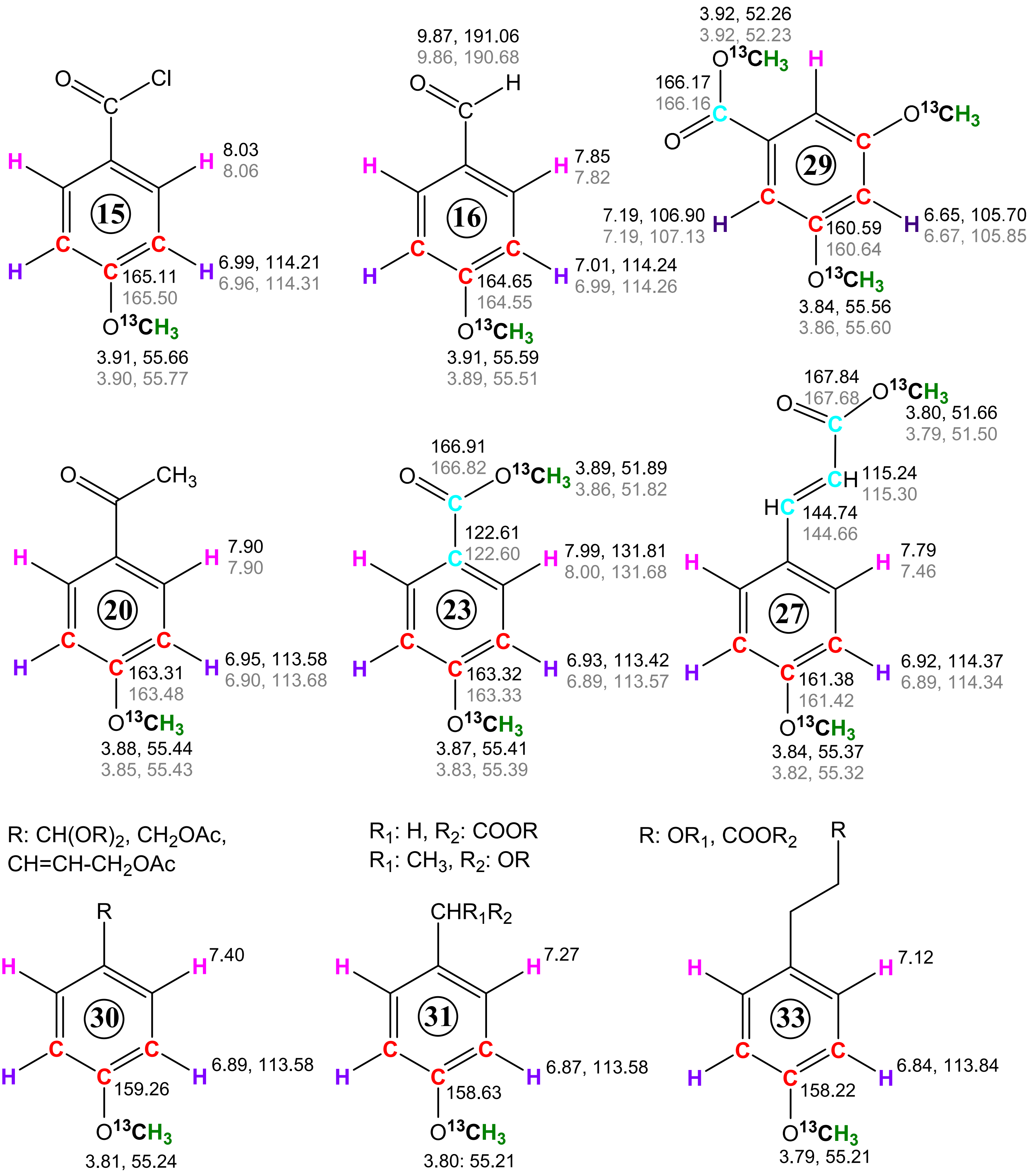
15
8.03
8.06
6.99, 114.21
6.96, 114.31
165.11
165.50
3.91, 55.66
3.90, 55.77
16
9.87, 191.06
9.86, 190.68
7.85
7.82
7.01, 114.24
6.99, 114.26
164.65
164.55
3.91, 55.59
3.89, 55.51
29
3.92, 52.26
3.92, 52.23
166.17
166.16
7.19, 106.90
7.19, 107.13
6.65, 105.70
6.67, 105.85
160.59
160.64
3.84, 55.56
3.86, 55.60
20
7.90
7.90
6.95, 113.58
6.90, 113.68
163.31
163.48
3.88, 55.44
3.85, 55.43
23
166.91
166.82
3.89, 51.89
3.86, 51.82
122.61
122.60
7.99, 131.81
8.00, 131.68
6.93, 113.42
6.89, 113.57
163.32
163.33
3.87, 55.41
3.83, 55.39
27
167.84
167.68
3.80, 51.66
3.79, 51.50
115.24
115.30
144.74
144.66
7.79
7.46
6.92, 114.37
6.89, 114.34
161.38
161.42
3.84, 55.37
3.82, 55.32
R: CH(OR)2, CH2OAc, CH=CH-CH2OAc
R1: H, R2: COOR
R1: CH3, R2: OR
R: OR1, COOR2
30
7.40
6.89, 113.58
159.26
3.81, 55.24
31
CHR1R2
7.27
6.87, 113.58
158.63
3.80: 55.21
33
7.12
6.84, 113.84
158.22
3.79, 55.21

R: $CH(OR)_2$, CH_2OAc,
$CH=CH-CH_2OAc$

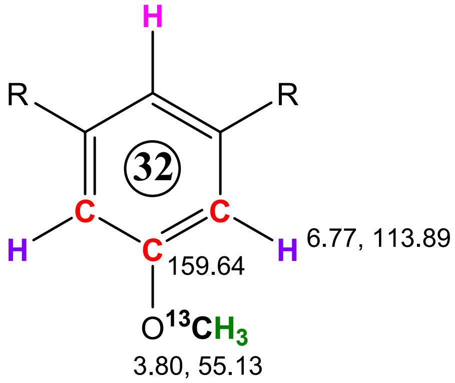

The theoretical chemical shifts of compound 4 were calculated by adding the effect of a $COOCH_3$ group in position 1 to the database values of methyl 2-methoxybenzoate.
